# A comprehensive assessment of post-discharge antibiotic use across an integrated healthcare system

**DOI:** 10.1017/ice.2025.10230

**Published:** 2025-11

**Authors:** Daniel J. Livorsi, Matthew Bidwell Goetz, Bruce Alexander, Brice Beck, Shylo E. Wardyn, Sarah C. Murray, Michihiko Goto

**Affiliations:** 1 Center for Access and Delivery Research and Evaluation, Iowa City Veterans Affairs Health Care System, Iowa City, IA, USA; 2 Division of Infectious Diseases, University of Iowa Carver College of Medicine, Iowa City, IA, USA; 3 VA Greater Los Angeles Healthcare System, Los Angeles, CA, USA; 4 David Geffen School of Medicine at the University of California, Los Angeles, CA, USA

## Abstract

**Objective:**

Antibiotics prescribed at hospital discharge are difficult to capture electronically for surveillance purposes unless patients are discharged to home with oral antibiotics. Our goal was to estimate the proportion of post-discharge antibiotics that are administered either in post-acute care facilities or via outpatient parenteral antibiotic therapy (OPAT) programs among patients discharged from Veterans Health Administration (VHA) hospitals.

**Design:**

We performed a retrospective study of all acute-care VHA admissions discharged during 2018–2021. The primary outcome was post-discharge antibiotic length of therapy, defined as the number of days of antibiotic exposure prescribed or recommended by inpatient providers at hospital discharge. Data on post-discharge antibiotic use was measured electronically for some discharge locations and estimated by performing manual chart reviews in randomly selected cases discharged to other locations.

**Setting:**

129 VHA hospitals.

**Results:**

There were 1,972,940 admissions, and 42.6% received inpatient antibiotics; 89.8% of patients were discharged to the community and 10.2% to post-acute care. The frequency of receiving post-discharge antibiotics varied by discharge location. Based on our calculations, 32.8% of all post-discharge days of antibiotic exposure occurred in post-acute care or via OPAT. Overall, 43.9% of all hospital-associated days of antibiotic exposure were administered during the hospital stay and the remaining 56.1% qualified as post-discharge.

**Conclusions:**

A third of all post-discharge antibiotics were dispensed in post-acute care facilities or by OPAT programs. These findings have implications for comparing hospitals on their post-discharge antibiotic use, because antibiotic data for patients discharged to these locations is often missing or difficult to collect.

## Introduction

Approximately half of all patients are exposed to antibiotics during their hospital stay,^1^ and antibiotics are continued in a substantial proportion of these patients at the time of hospital discharge.^2^ These post-discharge antibiotics, which may be overlooked by antibiotic stewardship programs, are frequently unnecessary or sub-optimal.^3–6^

Prior studies have tried to estimate the proportion of all antibiotics associated with a hospital stay that qualify as post-discharge. A study in the Veterans Health Administration (VHA) found that, during 2014–2016, 40% of the total duration of antibiotic exposure occurred after hospital discharge.^2^ This study only captured oral antibiotics prescribed at hospital discharge, typically in patients who were discharged to home. In non-VHA hospitals, data on intended post-discharge antibiotics can be captured from electronic discharge prescriptions, or e-scripts.^7^

A major limitation of these prior studies is that they were unable to collect data on antibiotics that were recommended by inpatient providers but were (a) given to patients in post-acute care facilities or (b) administered via outpatient parenteral antibiotic therapy (OPAT) programs. In both these situations, the post-discharge antibiotics are not electronically prescribed or dispensed by the acute-care hospital and therefore more difficult to collect. It is unclear how much of all post-discharge antibiotics are administered in these settings.

The VHA is the largest integrated healthcare system in the US and provides a unique opportunity to study post-discharge antibiotic use given its fairly comprehensive data on both inpatient and outpatient antibiotic-prescribing. The purpose of this study was to estimate the proportion of post-discharge antibiotics that are administered either in post-acute care facilities or via OPAT across VHA hospitals.

## Methods

We performed a retrospective study across all acute-care admissions within the VHA system during 2018–2021. The institutional review board (IRB) of the University of Iowa and Iowa City Veterans Health Care System approved this study. Waiver for informed consent was granted by the IRB for this retrospective cohort.

### Data sources

We accessed national administration data from the VHA Corporate Data Warehouse (CDW) via the VHA Informatics and Computing Infrastructure. This included data on patient demographics, comorbidities, inpatient procedures, primary services, and discharge diagnoses of infection. Antibiotic use was identified in the inpatient Barcode Medication Administration pharmacy data domain of the CDW, the outpatient medication files, and through manual chart reviews done by two trained abstractors (S.E.W., S.C.M.) using the Compensation and Pension Record Interchange. We collected data on all antibacterials (hereafter “antibiotics”) included in the National Healthcare Safety Network’s Antimicrobial Use and Resistance Protocol.^8^

### Inclusion criteria

We included patient-admissions if they were admitted during 2018–2021 to an acute care bed in any VHA medical center, including an intensive care unit, step-down unit, medical or surgical unit or observational bed. Patient admissions were excluded if their discharge status was classified as either (1) death, (2) transfer to another acute-care hospital, or (3) discharged to an unknown location.

### Outcomes

The primary outcome was post-discharge antibiotic length of therapy. Post-discharge length of therapy is the number of days of antibiotic exposure prescribed or recommended at hospital discharge, regardless of the number of agents prescribed per day. When measuring this outcome, we focused on both oral and intravenous (IV) antibiotic therapy either prescribed or recommended by inpatient providers for patients who were discharged to the community (e.g., home), non-VHA post-acute care, or VHA post-acute care. All post-discharge antibiotic prescriptions were truncated at 42 days to avoid counting indefinite antibiotic suppression or prophylaxis.

Antibiotics prescribed and dispensed by the VHA were captured electronically. In situations when electronic data on post-discharge antibiotics was not available, we manually collected recommended antibiotic therapy from discharge summaries and/or the final inpatient notes of hospital teams in a randomly selected subset of patients (Supplement 1–2). We assumed that the documented, or recommended, plan was shared with the post-acute care facility or OPAT program that would be administering the patient’s antibiotics after discharge.

Discharge locations were captured electronically, and based on manual chart reviews (see below), found to be accurately coded in ∼95% of cases. Discharge locations were classified as community (e.g., home), VHA post-acute care (e.g., Community Living Center, or CLC), and non-VHA post-acute care (e.g., community nursing home). Prior work has shown that CLCs and non-VHA post-acute care facilities manage markedly different patient populations.^9^

Post-discharge oral antibiotics were captured both electronically and through manual chart reviews. For patients discharged to the community (e.g., home), we electronically captured post-discharge oral antibiotics—all of which were dispensed from the VHA outpatient pharmacy during the discharge period.^2^ For patients transferred to VHA post-acute care who were on inpatient antibiotics on the day of hospital discharge and/or the day before discharge, we electronically captured oral antibiotics that were administered on day 1 of their post-acute care stay and, if relevant, continued on consecutive days thereafter. Only patients who exclusively received oral antibiotics without concomitant IV antibiotics were classified as receiving oral therapy. Electronic data on post-discharge oral antibiotics was not available for patients transferred to a non-VHA post-acute care facility. To estimate the proportion of patients discharged on oral antibiotics to non-VHA post-acute care, we performed manual chart reviews of 404 randomly-selected patients transferred to non-VHA post-acute care who were on any inpatient antibiotics on the day of hospital discharge and/or the day before discharge (Supplement 1). These manual chart reviews were performed across 20 VHA hospitals, i.e., 5 facilities of varying complexity within each US Census region (Northeast, South, Midwest, and West). Manual reviews captured the duration of post-discharge oral antibiotics recommended by the VHA inpatient providers. Data on the actual duration of post-discharge antibiotics administered to the patient in non-VHA post-acute care was not available in VHA records.

Post-discharge IV antibiotics were also captured both electronically and through manual chart reviews. For patients transferred to VHA post-acute care who were on inpatient IV antibiotics on the day of hospital discharge and/or the day before discharge, we electronically captured IV antibiotics that were administered on day 1 of their post-acute care stay and, if relevant, continued on consecutive days thereafter. Patients who received both IV and oral antibiotics concomitantly were classified as receiving an IV-based regimen. Electronic data on post-discharge antibiotics was not available for patients who were on OPAT and were discharged either to the community or to a non-VHA post-acute care facility. To estimate the proportion of patients who were discharged to these two locations on OPAT, we performed manual chart reviews in 500 randomly-selected patients from across all VHA hospitals who met specific electronic criteria: (a) on IV antibiotics on the day of discharge and/or the day before discharge; (b) had a discharge diagnosis of a biliary infection, an intra-abdominal infection, a central nervous system infection, a complicated pneumonia (e.g., empyema or abscess), endocarditis, or an osteoarticular infection—or—had a positive microbiologic culture from the blood, synovial fluid or cerebrospinal fluid (Supplement 2), and (c) had an inpatient Infectious Disease (ID) consultation during the index hospital stay. The rationale for criterion (c) was that, based on a preliminary review of 145 OPAT patients, nearly all had a documented recommendation from the ID consultation service. These manual chart reviews captured the duration of post-discharge IV antibiotics recommended by the VHA inpatient providers. Data on the actual duration of post-discharge antibiotics administered via OPAT was often not available.

### Statistical analysis

Descriptive statistics were summarized with medians, interquartile ranges, percentages, means, and standard deviations. We used the findings from our manual chart reviews to estimate the percentage of patients with a specific disposition who received post-discharge antibiotics; we constructed 95% confidence intervals around each estimate using a binomial “exact” calculation.^10,11^ Data on duration of post-discharge antibiotics collected through manual chart reviews were used to estimate post-discharge antibiotic durations in patients with that same disposition.

## Results

There were 2,060,678 admissions across 129 hospitals during 2018–2021, of which 43,142 (2.1%) were transferred to another acute-care hospital, 34,455 (1.7%) died while hospitalized, and 10,141 (0.5%) were discharged to an unknown location. Therefore, 1,972,940 patient-admissions were included in the final study cohort (Table 1). The median age of included patients was 70 years (IQR 61–75).

**Table 1.** Characteristics of patients discharged to the community or to post-acute care from VHA hospitals, 2018–2021

	Total *n* = 1,972,940	Discharged to the community *n* = 1,771,096	Non-VHA post-acute care *n* = 70,314	VHA post-acute care *n* = 131,530
**Age**, median (IQR)	70 (61–75).	69 (61–75)	75 (69–84)	70 (61–76)
**Male sex**, n (%)	1,853,175 (93.9)	1,660,709 (93.8)	67,669 (96.2)	124,797 (94.9)
**Comorbidities**				
CHF	620,755 (31.5)	552,422 (31.2)	28,708 (40.8)	39,625 (30.1)
Cancer	467,380 (23.7)	421,530 (23.8)	16,780 (23.9)	29,070 (22.1)
COPD	754,858 (38.3)	674,458 (38.1)	29,624 (42.1)	50,776 (38.6)
Diabetes mellitus	894,262 (45.3)	797,708 (45.0)	37,372 (53.2)	59,182 (45.0)
ESRD	74,024 (3.8)	63,433 (3.6)	4,311 (6.1)	6,280 (4.8)
Liver disease	350,141 (17.8)	313,291 (17.7)	11,721 (16.7)	25,129 (19.1)
**Infection diagnoses**				
Biliary or intra-abdominal	74,022 (3.8)	67,788 (3.8)	2,183 (3.1)	4,051 (3.1)
CNS infection	5,294 (0.3)	3,906 (0.2)	430 (0.6)	958 (0.7)
Endocarditis	14,354 (0.7)	10,878 (0.6)	1,051 (1.5)	2,425 (1.8)
Osteoarticular	68,251 (3.5)	49,933 (2.8)	5,234 (7.4)	13,084 (10.0)
Pneumonia^1^	156,661 (7.9)	131,382 (7.4)	10,113 (14.4)	15,166 (11.5)
Skin-soft tissue	186,309 (9.4)	145,424 (8.2)	14,772 (21.0)	26,113 (19.9)
Urinary tract	161,535 (8.2)	128,303 (7.2)	14,018 (19.9)	19,214 (14.6)
**Length of hospital stay**, median (IQR) in days	3 (1–5)	3 (1–5)	7 (4–12)	5 (2–9)
**Inpatient antibiotic exposure,** n (%)	840,877 (42.6)	731,575 (41.3)	41,578 (59.1)	67,724 (51.5)
**Inpatient antibiotic duration**, median (IQR)^2^	3 (2–6)	3 (2–5)	6 (3–9)	5 (3–9)
**Inpatient antibiotics just prior to discharge**, n (%)^3^	674,014 (34.2)	601,080 (33.9)	25,503 (36.3)	47,431 (36.1)

Note. CHF, congestive heart failure; CNS, central nervous system; COPD, chronic obstructive pulmonary disease; ESRD, end-stage renal disease and on dialysis; IQR, interquartile range; VHA, Veterans Health Administration.

1 This includes both uncomplicated pneumonia and pneumonia complicated by an empyema or abscess.

2 Inpatient antibiotic duration was limited to patients who received inpatient antibiotics.

3 These patients received inpatient antibiotics on the day of discharge or the day before discharge.

Inpatient antibiotic treatment was administered to 840,877 (42.6%) patient-admissions. The median duration of inpatient antibiotics was 3 days (IQR 2–6), and the total days of inpatient antibiotic exposure across all patients was 3,912,642.

There were 1,771,096 (89.8%) patients discharged to the community; 98.4% of these patients were discharged to home with self-care. Other patients discharged to the community were electronically classified as receiving home health (0.9%), home hospice (0.4%), or being placed in another location in the community that was not a private residence (0.3%).

There were 201,844 (10.2%) patients discharged to post-acute care, including 70,314 (3.6%) transferred to non-VHA post-acute care and 131,530 (6.7%) to VHA post-acute care. The most common destinations in VHA post-acute care were a Community Living Center (83.4%), which is similar to a skilled nursing facility, followed by mental health (8.2%), rehabilitation (5.1%), and other (3.4%).

### Post-discharge oral antibiotics

Post-discharge oral antibiotics were prescribed to 354,240 (20.0%) of the 1,771,096 patients discharged to the community for a mean duration of 9.5 days (SD 9.4) (Table 2); 23,403 (6.6%) of these patients had not received any inpatient antibiotics. Of the 601,080 patients discharged to the community who were on inpatient antibiotics on the day of discharge or the day before discharge, 54.4% received post-discharge antibiotics.

**Table 2.** The frequency and duration of post-discharge antibiotics recommended or prescribed by VHA hospitals (2018–2021), based on the patient’s discharge location

	Discharge Location		
	Discharged to the community *n* = 1,771,096	Non-VHA post-acute care *n* = 70,314	VHA post-acute care *n* = 131,530
**Post-discharge oral antibiotics prescribed**, %	20.0%	21.8%^2^	15.9%
**Post-discharge oral antibiotic duration**, median days (IQR)^1^	7 (4–10)	5 (3–11)^2^	7 (4–15)
**Post-discharge IV-based antibiotic regimen prescribed, %**	1.7%^3^	5.7%^3^	14.2%
**Post-discharge IV-based regimen duration**, median days (IQR)^1^	28 (14–38)^3,4^	26 (14–36)^3^	12 6–28)

1 Post-discharge antibiotic duration was calculated only for patients who received post-discharge antibiotics.

2 This represents an estimate based on 404 manual chart reviews. The 95% confidence interval around this point estimate of 21.8% was 20.0–23.6%.

3 This represents an estimate based on 500 manual chart reviews. The 95% confidence interval around the point estimate for the community (1.7%) was 1.5–1.9%. For non-VHA post-acute care, the 95% confidence interval around the point estimate (5.7%) was 5.1–6.3%.

4 Antibiotic duration for patients discharged to the community may have been counted twice among patients who received both oral and IV post-discharge antibiotics. This is because the electronic data on post-discharge oral antibiotics did not indicate if patients also received post-discharge IV antibiotics. In the 192 patients who underwent manual chart review and were discharged to the community on IV antibiotics, only 35 (18.2%) also received oral antibiotics. Therefore, the effect of double-counting should be small.

Among the 70,314 patients transferred to non-VHA post-acute care, there were 25,503 (36.3%) who were on antibiotics on the day of hospital discharge or the day before hospital discharge. Based on manual chart reviews in 404 of these patients, 243 (60.2%; 95% CI 55.2–65.0%) were discharged to non-VHA post-acute care on an exclusively oral antibiotic regimen. The mean recommended duration of oral antibiotics after discharge in these patients was 11.4 days (SD 14.0). Based on these findings, the estimated percentage of all patients transferred to non-VHA post-acute care on oral antibiotics was 21.8% (95% CI, 20.0–23.6%).

Post-discharge oral antibiotics were prescribed to 20,962 (15.9%) of the 131,530 patients transferred to VHA post-acute care (Table 2); the mean duration of these exclusively oral antibiotic regimens was 11.7 days (SD 11.7). Of the 47,431 patients transferred to VHA post-acute care who were on inpatient antibiotics on the day of discharge or the day before discharge, 44.2% received post-discharge oral antibiotics.

### Post-discharge IV antibiotics

There were 66,305 patients discharged to the community or non-VHA care who met our electronic criteria for possibly receiving OPAT, which consisted of 61,113 patients discharged to the community and 5,192 transferred to non-VHA post-acute care; 279 of the 500 manually reviewed cases actually received OPAT (55.8%; 95% CI 51.3–60.2%), including 192 (49.5%; 95% CI 44.4–54.6%) of the 388 patients who were discharged to the community and 87 (77.7%; 95% CI 68.8–85.0%) of the 112 patients who were transferred to non-VHA post-acute care. Based on these findings, the estimated percentage of patients discharged to the community or transferred to non-VHA post-acute care on OPAT across all discharges to these locations was 1.7% (95% CI, 1.5–1.9%) and 5.7% (95% CI, 5.1–6.3%), respectively (Table 2). The mean duration of an IV-based regimen in patients discharged to the community was 26.3 days (SD 13.2), while the mean duration of an IV-based regimen in patients transferred to post-acute care was 24.8 days (SD 13.2).

The most common infection treated with OPAT in the manually reviewed cases was osteoarticular infections, which was the indication for antibiotics in 161 (57.7%) OPAT patients. Further description of OPAT indications and therapy is shown in Supplement 3.

Of the 131,530 patients transferred to VHA post-acute care, there were 18,663 (14.2%) who were on IV antibiotics just before hospital discharge and were continued on an IV-based regimen after transfer (Table 2). The mean duration of these post-discharge IV-based antibiotic regimens was 17.2 days (SD 13.9).

### Estimating the proportion of post-discharge antibiotics administered in post-acute care or via OPAT

Table 3 shows the actual or estimated number of post-discharge antibiotic days during 2018–2021 based on route of administration and discharge location. Based on our calculations, 32.8% of all post-discharge antibiotics were administered in post-acute care or via OPAT. Overall, 43.9% of all hospital-associated days of antibiotic exposure were administered during the hospital stay and the remaining 56.1% qualified as post-discharge. An estimated 4,153,630 (83.1%) of post-discharge days were administered in the community while 842,751 (16.9%) were administered in post-acute care, either within or outside of VHA.

**Table 3.** Total post-discharge antibiotic days (measured or estimated) for patients discharged from VHA hospitals, 2018–2021

*Disposition location*	Total antibiotic days
(1) Outpatient parenteral antibiotic therapy (OPAT) for patients discharged to the community or transferred to non-VHA post-acute care	894,394*
(2) Oral antibiotics in patients discharged to non-VHA post-acute care	175,432*
(3) VHA post-acute care (oral and IV antibiotics)	567,485
(4) Oral outpatient antibiotics for patients discharged to the community	3,359,070
Total post-discharge antibiotic days	4,996,381
% of post-discharge antibiotics given in post-acute care and/or via OPAT	32.8%

* Estimation based on manual chart review findings, as outlined in the Methods. For row 1, there were an estimated 794,560 in patients discharged to the community and 99,834 antibiotic days in patients who were transferred to non-VHA post-acute care.

### Intra-facility variation in disposition location

There was inter-hospital variation in whether patients were discharged to these settings. At the hospital-level, the median frequency of patients meeting our electronic criteria for possible OPAT was 3.5% (IQR 2.2–4.3%). In addition, at the hospital-level, the median frequency of discharging a patient who was on antibiotics just prior to discharge to post-acute care was 4.1% (IQR 2.6–5.5%).

## Discussion

In this retrospective cohort of acute-care admissions from 129 hospitals in the VHA system, we found that a third of all post-discharge days of antibiotic exposure were dispensed in post-acute care facilities or by OPAT programs. These findings have implications for comparing hospitals on their post-discharge antibiotic use, because post-discharge antibiotic data for patients discharged to these locations is often missing or difficult to collect.

In our prior work, we proposed a metric for comparing hospitals on their post-discharge antibiotic use.^12^ This metric excluded patients who were discharged to post-acute care and acknowledged that patients receiving OPAT could not be captured. This current study demonstrates that a large proportion of a hospital’s post-discharge antibiotic use will be not evaluated by the current design of the metric.

By leveraging both electronic data and targeted manual chart reviews, we estimated that more than half of antibiotics associated with an acute-care hospital stay are used after the patient left the hospital. This estimate includes a combination of post-discharge antibiotics actually prescribed by the inpatient providers and post-discharge antibiotics that the inpatient providers recommended for post-acute care facilities to administer to the patient. Prior studies, which were incomplete in their data collection, reported that post-discharge antibiotics accounted for about 40% of total hospital-associated days of antibiotic exposure.^2,7^

Our findings again highlight the potential opportunity for antibiotic stewardship around hospital discharge. Depending on the patient’s discharge location, we found that roughly half of patients on inpatient antibiotics immediately prior to discharge went on to receive post-discharge antibiotics. This suggests that the time just before discharge could be a moment for a well-designed stewardship process to prospectively identify patients likely to receive post-discharge antibiotics. Several non-randomized trials have shown that auditing and providing feedback at discharge can improve antibiotic-prescribing at this transition of care.^5,13–17^ For example, a pharmacist-driven practice model across 5 Michigan hospitals demonstrated success at improving post-discharge antibiotic use by identifying patients who were still on inpatient antibiotics as they approached discharge and then working with the primary teams to optimize antibiotic-prescribing.^17^ It would be valuable to replicate these findings in other healthcare systems that have different inpatient antibiotic stewardship resources and processes.

One strength of our study is our relatively robust approach to estimating post-discharge antibiotic use in locations where electronic data was not available. We were able to electronically capture post-discharge antibiotics administered in post-acute care locations within the VHA. In contrast, most health systems would not have access to similar data from the post-acute care facilities where their patients are transferred. Replicating our findings outside of the VHA system would therefore be challenging.

Our study also has some limitations that should be acknowledged. First, our analysis only captured the duration of post-discharge antibiotics that was recommended or prescribed, not what was actually administered or taken by the patient. Second, when identifying cases for manual chart review to measure the use of OPAT, we may have overlooked cases that did not have an ID consultation. This could be one explanation for why we measured differences in the frequency of post-discharge IV antibiotic use between patients who were discharged to non-VHA (5.7%) versus VHA (14.2%) post-acute care. However, it is also worth noting that at least one prior study found higher rates of IV medication use (e.g. IV antibiotics) among Veterans in VHA versus non-VHA post-acute care facilities.^9^ Furthermore, because the cost of a patient’s care is less of a concern at post-acute care facilities within VHA, it is plausible that acute-care patients on IV antibiotics were more often accepted by post-acute care facilities in VHA versus outside of VHA. A third limitation is that the confidence intervals for our estimates of OPAT and oral post-discharge antibiotics in non-VHA post-acute care both span +/– 5%, so we may have either overestimated or underestimated the amount of post-discharge antibiotics administered in these locations. Fourth, we stopped counting post-discharge antibiotic duration at 42 days even if the prescription extended beyond that point. This cut-off was used to prevent counting antibiotic days for chronic suppression or prophylaxis, but some prescriptions longer than 42 days may have actually been for therapeutic purposes. On the other hand, we may have inadvertently counted some post-discharge courses intended for suppression or prophylaxis by not truncating courses sooner than 42 days. Fifth, our estimate of post-discharge antibiotics only reflects antibacterials, not antifungals or antivirals. Sixth, the COVID-19 pandemic occurred during our 4-year observation period, and antibiotic-prescribing practices at discharge may have deviated from the norm during this time frame. For example, discharging patients to post-acute care facilities was difficult early during the pandemic, and this may have affected our estimates of post-discharge antibiotic use in these locations. Finally, our findings from this study may not be generalizable to non-VHA settings.

In conclusion, our comprehensive assessment of post-discharge antibiotic use across the entire VHA found that a third of post-discharge antibiotics are dispensed via OPAT programs or in post-acute care facilities. By capturing post-discharge antibiotics administered in these locations, we estimate that more than half of all antibiotic days associated with a hospital stay are taken by the patient after hospital discharge. Given the large volume of antibiotic use at this transition of care, further studies to evaluate and improve antibiotic-prescribing at hospital discharge are warranted.

## Supporting information

Livorsi et al. supplementary material Livorsi et al. supplementary material

## Supplementary material

To view supplementary material for this article, please visit https://doi.org/10.1017/ice.2025.10230
