# Supplementary material for: A comprehensive assessment of post-discharge antibiotic use across an integrated healthcare system
Source: Infect Control Hosp Epidemiol. 2025 Sep 17;46(11):1134–9. doi: 10.1017/ice.2025.10230 (PMC12620061; doi:10.1017/ice.2025.10230)
Supplement: Livorsi et al. supplementary material [file S0899823X25102304sup001.docx]

**Supplement 1. Approach to manual chart reviews to estimate oral antibiotic use among patients transferred to non-VHA post-acute care**

**Inclusion criteria:**

1. Discharged from a VHA acute-care location between January 1, 2018 and December 31, 2021;
2. Alive at the time of discharge;
3. Place of disposition electronically categorized as either "Hospice" or " Nursing Home, non-VHA."
4. Received an inpatient antibiotic (either intravenously or by mouth) on the day before discharge and/or the day of discharge

**Selection of VHA hospitals to review**

- Each VHA acute-care facility was assigned to one of the four US Census Regions (West, South, Northeast and Midwest).
- For each of the 4 regions, we randomly selected two 1a complexity facilities. Facility complexity scores are created by the VHA Healthcare Analysis and Information Group. Hospitals are scored according to their patient population, clinical services (e.g., intensive care unit and surgery services), education and research. A score of 1a is the most complex while a score of 3 is the least complex.
- For each of the 4 regions, we randomly selected one facility from each of the following complexity categories: 1b, 1c, and 2
- Target enrollment across all 20 hospitals was 420 admissions, which included the following numbers of discharges per facility. All qualifying admissions were randomly selected for manual chart review:
  - 25 qualifying patients from each level 1a facility
  - 20 qualifying patients from each level 1b and 1c facility
  - 15 qualifying patients from each level 2 facility

In total, 420 patients underwent manual chart review: 16 patients electronically classified as discharges to “Hospice” and 404 electronically classified as discharges to " Nursing Home, non-VHA."

- 15 of 16 (93.8%) patients discharged to hospice were discharged to home hospice;
- 384 of the 404 (95.0%) discharges to a non-VHA nursing home actually were discharged to a post-acute care facility outside VHA, such as a rehabilitation facility or a skilled nursing facility.

Findings on antibiotic use in these patients are described in the manuscript.

**Sample size calculation**: We estimated that 65% of patients who met the inclusion criteria would be discharged on oral antibiotics. To achieve a confidence interval that extended no more than +/- 5% around our point estimate, we determined that at least 353 chart reviews were required.

**Supplement 2. Approach to manual chart reviews to estimate intravenous antibiotic use among patients discharged to the community or transferred to non-VHA post-acute care**

**Inclusion criteria:**

1. Discharged from a VHA acute-care location between January 1, 2018 and December 31, 2021;
2. Alive at the time of discharge;
3. Place of disposition electronically categorized as either "Home," "Community, not home,” “Home Health, or “Nursing home, non-VHA.";
4. Received an inpatient intravenous antibiotic on the day before discharge and/or the day of discharge;
5. An Infectious Disease consultation, either in-person or electronic, was performed during the patient’s hospital stay;
6. A blood culture, synovial fluid culture, or cerebrospinal fluid culture (collected within 24 hours before admission through the date of hospital discharge) grew a likely bacterial pathogen and/or the patient had a discharge diagnosis for one of the following infections, based on International Classification of Diseases, 10^th^ revision codes (ICD-10):
   - - Biliary tract infection
     - Central nervous system infection, such as meningitis and brain abscess
     - Endocarditis or other endovascular infection (e.g., graft infection)
     - Intra-abdominal infection
     - Osteo-articular infection, including osteomyelitis and septic arthritis
     - Pneumonia with abscess and/or empyema
     - Pneumonia that underwent pleural drainage
     - Miscellaneous conditions, including diagnostic codes that indicated specific bacterial pathogens; syphilis and other sexually transmitted diseases; ophthalmologic infections; malignant otitis externa; gynecologic infections; line infections; infections related to procedures and medical devices; transplant-related infections

**Selection of cases to review:**

- 1. Cases that met the above electronic criteria were randomly selected from a national cohort that included all VHA facilities.
  2. Five hundred eligible cases were reviewed by two separate reviewers (i.e., 250 cases per reviewer).
  3. Twenty-two cases (4.4%) were actually transferred to non-qualifying location: another hospital (n=12), VHA post-acute care (n=9) or death (n=1).

Findings on antibiotic use in these patients are described in the manuscript and in Supplement 3.

**Sample size calculation**: We estimated that 65% of patients who met the inclusion criteria would be discharged on IV antibiotics. To achieve a confidence interval that extended no more than +/- 5% around our point estimate, we determined that at least 353 chart reviews were required. Because we wanted an adequate sample of both patients discharged to the community and patients discharged to a non-VHA post-acute care facility, we decided to review 500 charts in total.

**Supplement 3. Findings from manual chart reviews to estimate the frequency and duration of post-discharge intravenous antibiotic use among patients discharged to the community or transferred to non-VHA post-acute care**

**Frequency of intravenous (IV) antibiotic use based on discharge location**

- 192 of 388 patients (49.5%) electronically classified as being discharged to the community received post-discharge IV antibiotics
  - Mean duration of the IV-based antibiotic regimen: 26.27 days (SD 13.22)
  - Median duration of the IV-based antibiotic regimen: 28 days (14-38)
- 87 of 112 (77.7%) patients electronically classified as being discharged to post-acute care outside VHA received post-discharge IV antibiotics
  - Mean duration of the IV-based antibiotic regimen: 24.75 days (SD 13.24)
  - Median duration of the IV-based antibiotic regimen: 26 days (14-36)

**Vascular access**

- PICC line 245 (87.8%)
- Midline 17 (6.1%)
- Chest port or tunneled catheter 6 (2.2%)
- Peripheral IV 6 (2.2%)
- Dialysis access 4 (1.4%)
- Intramuscular injections 1 (0.4%)

**Type of infection***

- **Osteoarticular infection**: 161 (57.7%)
  - Osteomyelitis of a limb (e.g., metatarsal or phalanx): 90 (32.3%)
  - Septic arthritis, native joint: 19 (6.8%)
  - Vertebral osteomyelitis and/or epidural abscess: 19 (6.8%)
  - Prosthetic joint infection: 17 (6.1%)
  - Osteomyelitis of other body sites (e.g., pelvis, skull, sternum) 16 (5.7%)
- **Intra-abdominal or biliary infection:** 31 (11.1%)
- **Endovascular infection**, including endocarditis or graft infection: 22 (7.9%)
- **Bacteremia, primary**: 20 (7.2%)
- **Skin and soft tissue infection, complicated**: 17 (6.1%)
- **Central nervous system infection**: 9 (3.2%)
- **Urinary tract infection,** including acute bacterial prostatitis or bacteremia: 11 (3.9%)
- **Pulmonary infection**: 8 (2.9%)

**Staphylococcus aureus* bacteremia was present in 37 (13.3%)

**Number of IV agents prescribed:**

- One 233 (83.5%);
- Two 44 (15.8%);
- Three 2 (0.7%)
